# DNA Damage Response Differentially Affects BoHV-1 Gene Transcription in Cell Type-Dependent Manners

**DOI:** 10.3390/biomedicines10092282

**Published:** 2022-09-14

**Authors:** Linke Tang, Weifeng Yuan, Shitao Li, Xiuyan Ding, Liqian Zhu

**Affiliations:** 1 Institute of Life Science and Green Development, College of Life Science, Hebei University, Baoding 071002, China; 2 Institute of Animal Sciences, Chinese Academy of Agricultural Sciences, Beijing 100193, China; 3 Department of Microbiology and Immunology, Tulane University, New Orleans, LA 70118, USA; 4 Key Laboratory of Microbial Diversity Research and Application of Hebei Province, College of Life Science, Hebei University, Baoding 071002, China; 5 College of Veterinary Medicine, Yangzhou University, Yangzhou 225009, China

**Keywords:** bovine herpesvirus 1, DNA damage, DNA damage response, γH2AX, ultraviolet

## Abstract

Bovine herpesvirus 1 (BoHV-1), an important pathogen of cattle, is also a promising oncolytic virus. Recent studies have demonstrated that the virus infection induces DNA damage and DNA damage response (DDR), potentially accounting for virus infection-induced cell death and oncolytic effects. However, whether the global DDR network affects BoHV-1 productive infection remains to be elucidated. In this study, we show that global DDR induced by ultraviolet (UV) irradiation prior to BoHV-1 infection differentially affected transcription of immediate early (IE) genes, such as infected cell protein 0 (bICP0) and bICP22, in a cell-type-dependent manner. In addition, UV-induced DDR may affect the stabilization of viral protein levels, such as glycoprotein C (gC) and gD, because the variation in mRNA levels of gC and gD as a consequence of UV treatment were not in line with the variation in individual protein levels. The virus productive infection also affects UV-primed DDR signaling, as demonstrated by the alteration of phosphorylated histone H2AX (γH2AX) protein levels and γH2AX formation following virus infection. Taken together, for the first time, we evidenced the interplay between UV-primed global DDR and BoHV-1 productive infection. UV-primed global DDR differentially modulates the transcription of virus genes and stabilization of virus protein. Vice versa, the virus infection may affect UV-primed DDR signaling.

## 1. Introduction

Bovine herpesvirus 1 (BoHV-1), an important pathogen of cattle, is an enveloped DNA virus, belonging to the family Herpesviridae and the subfamily Alphaherpesvirinae [1,2]. The virus infection is an important cofactor for the induction of bovine respiratory disease complex (BRDC), which may result in life-threatening pneumonia, one of the most severe respiratory diseases in cattle [3]. BoHV-1 infection leads to suppression of the immune response and erosions of mucosal surface, which may cause secondary infection by polymicrobial pathogens, such as *Mannheimia haemolytica* (*MH*), a commensal bacterium of the upper respiratory tract in cattle. Consequently the viral–bacterial synergy induces pneumonia in most BRDC cases [4,5]. Apart from upper respiratory tract disorders and conjunctivitis, the virus infection is also associated with genital disease and late-term abortions in bovine species [6,7]. Furthermore, BoHV-1 is a major cause of viral abortion, with abortion rates ranging from 25% to 60% in cows [8]. Latency is generally established primarily in sensory neurons in the trigeminal ganglia (TG) of cows following acute infection. It can be periodically reactivated by diverse stressful stimuli, such as dramatic weather changes, long-distance shipping of cattle, and application of drugs, such as synthetic corticosteroid dexamethasone, which consequently leads to disease occurrence and virus shedding. BoHV-1 causes great economic burden to cattle herds worldwide [8]. Studies have indicated that BoHV-1 infection in cattle costs approximately USD three billion annually in the US [3].

Cellular DNA is vulnerable to the insult of many physical and chemical reagents, such as reactive oxygen species (ROS) (products of the cellular metabolism), ionizing radiation, and ultraviolet (UV) light, consequently leading to DNA damage. In addition, DNA damage may occur as a consequence of normal cellular processes, such as DNA replication and mitosis, as reviewed by [9,10]. The resulting DNA lesions, particularly DNA double-strand breaks (DSBs), tend to induce genomic instability and disease development [11,12]. As a nuclear replicating virus, BoHV-1 infection induces oxidative DNA damage in multiple cell cultures, such as bovine kidney cells (MDBK) and human A549 lung carcinoma epithelial cells, as demonstrated by the increased production of DSBs analyzed by comet assay [10,13,14]. Accumulating studies have indicated that damaged DNA can activate various cell death pathways [13], and BoHV-1infection induces diverse forms of cell death both in vivo and in vitro [14,15,16,17,18,19], which underscores the implication of virus-infection-induced DNA damage in the virus pathogenesis. In addition, as an oncolytic virus, BoHV-1 infection in human lung adenocarcinoma cells A549 results in cell lesions partially by inducing DNA damage [10], which underlies the importance of DNA damage in the virus oncolytic effects.

In response to different types of DNA lesions, a network of cellular pathways, known as cellular DNA damage response (DDR), are mobilized, which sense, signal, and repair DNA lesions. Actually, DDR is essential to overcome the deleterious effects exerted by DNA damage, as reviewed by [20,21]. Among the activated DDR, the phosphorylation of the histone H2AX at Ser139 (γH2AX), an early event with specificity for DSBs, plays a central role in repairing DSBs, reviewed by [20]. We have recently reported that BoHV-1 infection in A549 cells induces formation of γH2AX foci and can be used as a hallmark of DNA damage [22], indicating that the DDR molecule γH2AX is mobilized during BoHV-1 productive infection. Apart from γH2AX, BoHV-1 interacts with some other components of the DDR network, such as 53BP1, structural maintenance of chromosome-1(SMC1), and ataxia telangiectasia mutated (ATM) Nijmegen breakage syndrome (NBS1) [23]. The disruption of ATM signaling promotes cell apoptosis in virus-infected MDBK cells [23]. Taken together, BoHV-1 infection induces DNA damage and concomitantly has differential effects on distinct DDR signaling with complicated mechanisms. However, whether the overall DDR network affects BoHV-1 productive infection has not been reported.

Here, we investigated the effects of overall DDR network or DNA damage induced by UV irradiation on viral productive infection in different cell cultures. For the first time, we provide evidence that UV-induced DDR prior to virus infection has differential effects on BoHV-1 viral gene expression with cell-type dependent manners. Vice versa, the virus infection has the capacity to adjust DDR signaling as demonstrated by alteration of γH2AX protein levels and formation of γH2AX foci induced by UV treatment prior to virus infection.

## 2. Materials and Methods

### 2.1. Cells and Virus

Both A549 and MDBK cells were obtained from the Chinese Model Culture Preservation Center (Shanghai, China). These cells were cultured in DMEM medium containing 10% fetal bovine serum (FBS) (Thermo Fisher Scientific, Waltham, MA, USA). BoHV-1,NJ-16-1 was isolated from cow semen in China [24]. The virus was propagated in MDBK cells in a large amount. Then, the aliquots were stored at −80 °C until use.

### 2.2. Antibodies

The sources of the antibodies used in this study were: phospho-H2A.X (Ser139) (γH2A.X) monoclonal antibody (mAb) (cat#2577), HRP (horseradish peroxidase)-labeled goat anti-mouse IgG (cat#7076), HRP-labeled goat anti-rabbit IgG (cat#7074), Cell Signaling Technology (Danvers, MA, USA); BoHV-1 gC mAb (cat#F2), BoHV-1 gD mAb (cat#1B8-F11), VMRD Inc. (Pullman, WA, USA); β-Actin mAb (cat#AC026), Abclonal; Alexa Fluor 488^®^-conjugated goat anti-rabbit IgG (H + L) (cat# A-11008), Invitrogen Life Technologies (Waltham, MA, USA).

### 2.3. Western Blot

Cell cultures of MDBK and A549 were subjected to either mock treatment/infection or UV irradiation/virus infection for designated time lengths. Then, the cell lysates were prepared by using the lysis buffer with the following recipe: 1% Triton X-100, 1 mM EDTA, 50 mM sodium chloride, 20 mM sodium fluoride,1 mM EGTA, 20 mM sodium pyrophosphate, 1 mM phenylmethylsulfonyl fluoride, 1 mM benzamidine, 0.5 g/mL leupeptin, and 1 mM sodium orthovanadate in 20 mM Tris-HCl, pH 8.0.

The cell lysates were clarified by centrifugation at 13,000× *g* for 10 min. Then, they were boiled in Laemmli sample buffer for 5 min and subjected to separation on SDS-PAGE (8% or 10%), and then transferred to polyvinylidene fluoride (PVDF) membranes. After blocking with 5% skim milk, the membranes were incubated with the indicated primary antibodies, including the antibodies against gC (1:3000 dilution), gD (1:3000 dilution), γH2A.X (1:2000), and H2A.X, followed by HRP-conjugated secondary antibodies. After extensive washing with TBS containing 0.05% Tween-20(TBST), the immune reactive bands were probed after enhanced chemiluminescence (ECL) reaction. As a protein loading control, β-Actin was probed in the same transferred membrane along with individual proteins. The band intensity, quantitatively analyzed with free Image J program (https://imagej.nih.gov/ij/download.html (accessed on 1 December 2020), was initially normalized to β-Actin, and the fold change after infection was calculated. Protein levels in mock-infected cells were arbitrarily set to 1.

### 2.4. Immunofluorescence Assay

A549 and MDBK cells of confluent in 2-well chamber slides (Nunc Inc., Weston, IL, USA) were exposed to UV irradiation or mock treatment for 4 min at room temperature to induce DNA damage and DDR. Then, the cells were infected with BoHV-1 (MOI = 0.1) for indicated time lengths. Then, the cells were fixed with 4% paraformaldehyde and permeabilized with 0.25% Triton X-100 for 10 min at room temperature, respectively, and blocked with 1% BSA in PBST for 1 h, followed by incubation with an antibody against γH2A.X (1:800 dilution) for 12 h at 4 °C. After washing three times with PBST, the cells were incubated with Alexa Fluor 488^®^-conjugated goat anti-rabbit IgG (H + L) (Invitrogen, cat# A-11008, Waltham, MA, USA) for 1 h in the dark. After washing three times, the nuclei were stained with 4ʹ,6- diamidino-2-phenylindole (DAPI). The cells were covered with coverslips using an antifade mounting medium (Electron Microscopy Sciences, Inc., cat# 50-247-04, Hatfield, PA, USA). Images were captured using a confocal microscope (Leica Camera, Wetzlar, Germany).

### 2.5. Quantification of mRNA by qRT-PCR

Confluent cells in 6-well plates, either mock-treated or treated with UV light for 4 min, were infected with BoHV-1. After infection for designated time lengths, total RNA was purified with TRIzol LS Reagent (Cat#10296010, Thermo Fisher Scientific, Waltham, MA, USA) following the manufacturers’ specifications. One µg of freshly prepared RNA was used as a template for the synthesis of the first-strand cDNA with commercial random hexamer primers by using Thermoscript™ RT-PCR system Kit (Cat#11146-024, Invitrogen, Waltham, MA, USA). The cDNA products were used as templates for relative qPCR to detect the mRNA levels of bICP0 and bICP22 with specific primers as described elsewhere [25], via the ABI 7500 fast real-time system (Applied Biosystems, Inc., Foster City, CA, USA). GAPDH mRNA was measured and used as an internal control to normalize gene expression. The data were analyzed using the equation 2−∆∆iz method by comparison to the control cells.

## 3. Results

### 3.1. Define a Condition That Can Efficiently Induce DNA Damage Response (DDR) in Cell Cultures

To investigate whether overall DDR network or DNA damage affects BoHV-1 productive infection, we used two cell cultures: MDBK cell, a bovine kidney cell line that is usually used to study the mechanisms of BoHV-1 lytic infection, and human lung adenocarcinoma cell line A549, which also supports the virus productive infection. Additionally, virus infection induces DNA damage in these cell cultures [13]. Here, UV exposure, a known efficient method to generate DSBs that subsequently triggers DDR [26,27], was employed to induce DDR prior to virus infection. Since UV-induced DNA damage is toxic to cell cultures, we initially define a condition that can efficiently induce DDR but not obvious cell death in both cell cultures. Here, we chose a short UV exposure duration of 4 min and a subsequent incubation period of 4 and 12 h for MDBK and A549 cells, respectively. 

**Figure 1:**
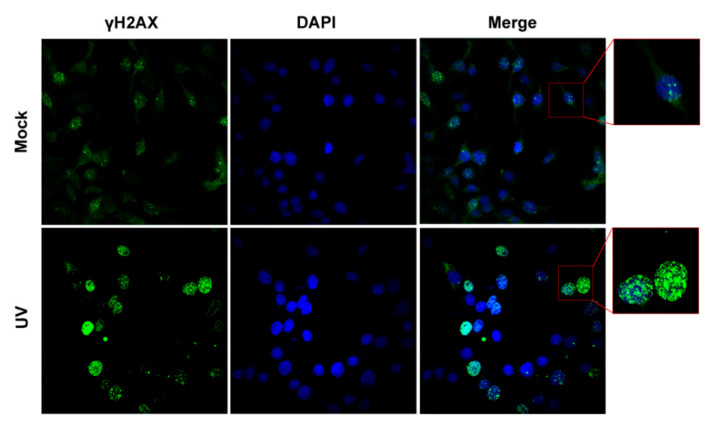
Detection of γH2AX foci in MDBK cells following UV treatment as an indicator of DDR. MDBK cells were either mock-treated or treated with UV light (40 W), attached to the hood (BIOBASE, Jinan, China), for 4 min, and subjected to the usual culture for 4 h. Then, γH2AX was detected by immunofluorescence (IFA). Nuclei were stained with DAPI. Images were obtained via confocal microscopy (magnification ×600). Framed cells were zoomed in showing representing γH2AX staining.

**Figure 2:**
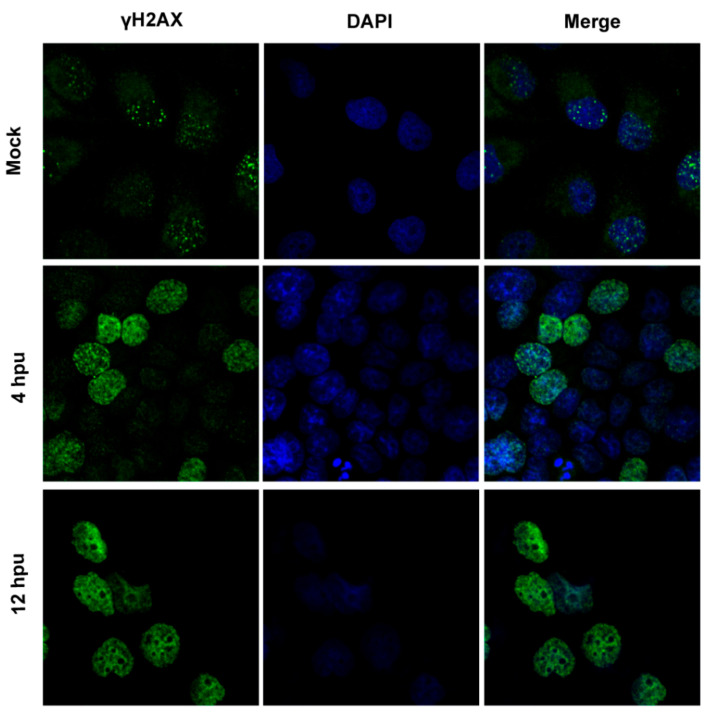
Detection of γH2AX foci in A549 cells following UV treatment as an indicator of DDR. A549 cells were either mock-treated or treated with UV light for 4 min and subjected to usual culture for 4 and 12 h. Then, IFA was performed to detect γH2AX. Nuclei were stained with DAPI. Images were obtained via confocal microscopy (magnification ×600).

To characterize whether DNA damage or DDR is induced within our conditions, IFA assay was performed to detect nuclear γH2AX foci, a hallmark of DDR. As expected, clear γH2AX foci were readily detected in mock-treated MDBK cells (Figure 1, upper panels), representing the basal levels of DDR. At 4 h post-UV treatment (hpu), MDBK cells harboring highlighted staining of γH2AX filling with the whole nucleus were readily detected in a subset of MDBK cells, where part of γH2AX established island-like structures rather than the speckle-like structures observed in mock-treated MDBK cells (Figure 1 zoomed-in area). Thus, it seems that UV treatment not only increased the number of γH2AX foci but also changed their morphology in a subset of MDBK cells. Then, IFA assay was performed in A549 cells, which received a UV exposure time of 4 min, then a subsequent culture for either 4 or 12 h to identify γH2AX foci. Relative to the mock-treated controls, fluorescence signaling of γH2AX is much higher in UV-treated A549 cells at both 4 and 12 hpu (Figure 2). Typical γH2AX foci with speckle-like morphology were observed in mock-treated A549 cells (Figure 2, upper panels). Unlike in mock-treated controls, the γH2AX foci with pleomorphism filled in the whole nucleus were observed in a population of cells at 4 hpu, while in some cells, the foci are indiscernible (Figure 2 middle panels). However, we found that γH2AX with highlighted staining without forming foci filled the whole nucleus at 12 hpu (Figure 2 bottom panels). It is possible that increased protein levels of nuclear γH2AX foci lead to fusion of the foci and consequently make them indistinguishable.

**Figure 3:**
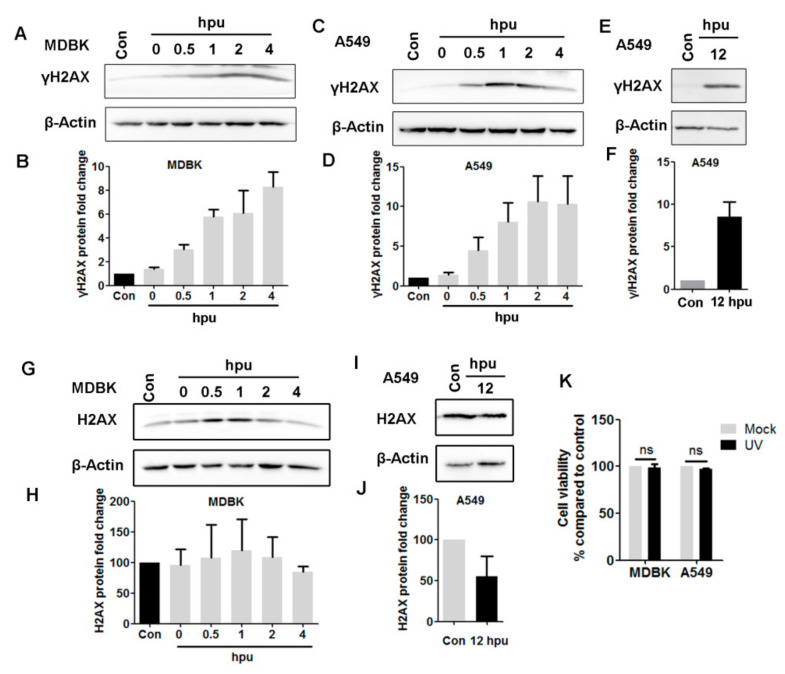
Detection of γH2AX protein levels following UV treatment as an indicator of DDR. Both MDBK (**A**) and A549(**C**,**E**,**I**) cells in 60 mm dishes were either mock-treated or treated with UV light for 4 min. Then, they were subjected to the usual culture for 4 (**A**,**C**,**G**) and 12 h (**E**,**I**). After washing three times with PBS, cell lysates were prepared for Western blot to detect both γH2AX and H2AX. β-Actin was probed and used as a protein loading control and subsequent quantitative analysis. Images represent data from three independent experiments. (**B**,**D**,**F**,**H**,**J**) Band intensity of either γH2AX or H2AX was initially normalized to β-Actin, and the fold change after UV treatment was calculated by comparison to that of mock-treated controls, which were arbitrarily set as 1 or 100%. (**K**) MDBK and A549 cells in 24-well plates were exposed to UV treatment for 4 min, then they were subjected to the usual culture for 4 and 12 h. Cell viability was detected by Trypan-blue exclusion test. The cell number of mock-treated controls was set as 100%.

Then, alterations in γH2AX protein levels were examined at various time points after UV treatment and subsequent culture for various time durations because an increased protein level of γ-phosphorylation of H2AX (γH2AX) is an early event in the DDR [20,28]. As a result, UV treatment increased γH2AX protein levels in MDBK cells at all the detected time points. Relative to that in mock-treated controls, the protein levels were increased approximately 3.03- and 8.31-fold at 0.5 and 4 hpu, respectively (Figure 3A,B). Similarly, UV treatment also increased the protein levels of γH2AX in A549 cells as detected from 0.5 to 12 hpu (Figure 3C,E), which were increased ranging from approximately 4.47- to 10.66-fold relative to the mock-treated controls (Figure 3B,D,F). The UV treatment did not obviously increase the protein levels of H2AX in MDBK cells (G and H), while it was decreased by approximately 50% relative to the mock-treated control in A549 cells as detected at 12 hpu (I and J). So, the increased phosphorylation of H2AX (γH2AX) in both cell cultures following UV treatment was not due to the increased protein levels of H2AX. UV treatment did not show evident cytotoxicity to these cell cultures at the indicated conditions in comparison to the mock-treated controls, as judged by the Trypan-blue exclusion test (Figure 3K), indicating that the increased phosphorylation of H2AX was not due to the affecting on the cell viability.

These data suggested that UV treatment in our conditions indeed increases the phosphorylation of H2AX, which corroborates our result observed in IFA assay as described in Figure 1 and Figure 2. Taking these data together, UV exposure within our conditions induces DDR, which satisfies our request for subsequent studies.

### 3.2. UV-Exposure-Induced DDR Has Differential Effects on Virus IE Transcription

**Figure 4:**
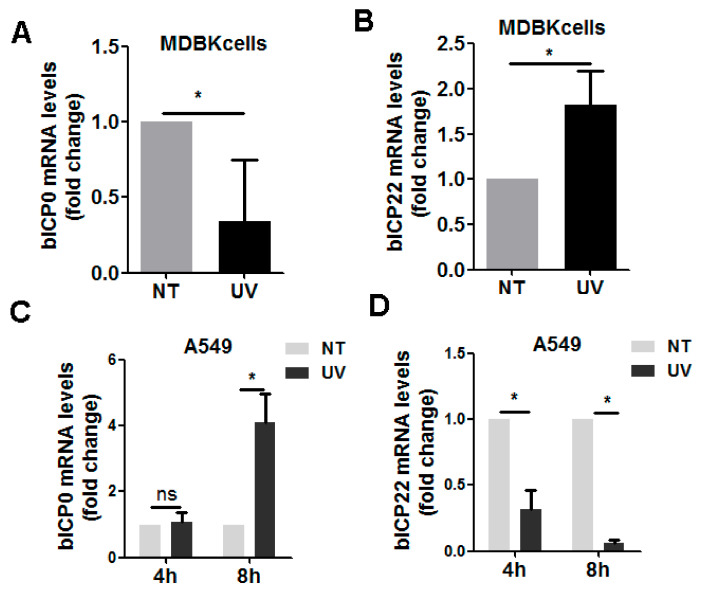
Effects of UV-induced DDR have on BoHV-1 mRNA expression of IE genes. (**A**,**B**) MDBK cells in 6-well plates were either mock-treated or treated with UV light for 4 min. After treatment they were infected with BoHV-1 (MOI = 0.1) for 4 h. Total RNA was purified, and subsequently mRNA levels of ICP0 (**A**) and ICP22 were detected by qRT-PCR. (**C**,**D**) A549 cells in 6-well plates either mock-treated or treated with UV light for 4 min were subsequently infected with BoHV-1 (MOI = 0.1) for 4 (**C**) and 8 (**D**) h, respectively. Then, total RNA was isolated, and mRNA levels of ICP0 and ICP22 were measured by qRT-PCR. The results shown are the mean of three independent experiments, with error bars indicating standard deviations. Significance was analyzed with Student’s *t*-test (* *p* < 0.05, ns, not significant).

During BoHV-1 productive infection in cell culture, virus gene expression is temporally regulated in three distinct phases: immediate early (IE), early (E), and late (L) [29]. Within our defined conditions, we initially investigated whether UV-induced DDR has effects on the expression of BoHV-1 immediate early (IE) genes. For this purpose, both cell cultures were exposed to UV irradiation for 4 min, followed by infection of 4 h in MDBK cells, as well as 4 and 8 h in A549 cells, respectively. Then, gene expression of viral regulatory proteins (bICP0) and bICP22 were examined with relative qRT-PCR. When DDR was induced prior to virus infection in MDBK cells, bICP0 mRNA levels were reduced to approximately 34.07% relative to the mock-treated control (Figure 4A). In contrast, after UV exposure, the bICP22 mRNA levels were approximately 1.82-fold higher than the mock-treated controls (Figure 4B). When DDR was induced prior to virus infection in A549 cells, bICP0 mRNA levels were increased approximately 4.12-fold at 8 hpi, but not affected at 4 hpi (Figure 4C), bICP22 mRNA levels were consistently reduced to approximately 32.09% and 6.52% relative to the control at 4 and 8 hpi, respectively. Taken together, the transcription of both bICP0 and bICP22 in both cell cultures was altered due to UV exposure prior to virus infection, which indicated that UV-induced DDR may have differential effects on virus IE expression with cell-type-dependent manners.

### 3.3. UV-Induced DDR Prior to Virus Infection Has Effects on the Stabilization of Vial Glycoproteins gC and gD

**Figure 5:**
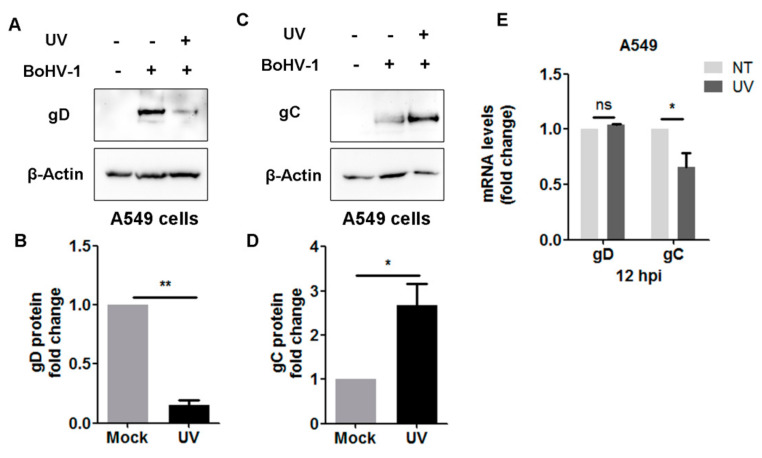
Effects of UV-induced DDR on the expression of viral protein gC and gD. (**A**,**C**) A549 cells in 60 mm dishes were either mock-treated or treated with UV light for 4 min, and subsequently infected with BoHV-1 (MOI = 0.1) for 12 h. After three washings with PBS, cell lysates were prepared for Western blot to detect gC and gD. Cellular protein β-Actin was probed as a protein loading control. Images represent data from three independent experiments. (**B**,**D**) The band intensity was initially normalized to β-Actin. The fold change induced by UV treatment was calculated by comparison to that of mock-treated controls, which were arbitrarily set as 1. (**E**) A549 cells in 6-well plates were either mock-treated or treated with UV light for 4 min, and subsequently infected with BoHV-1 (MOI = 0.1) for 12 h. Then, the RNA was extracted, and mRNA levels of gC and gD were detected by qRT-PCR. Results shown are the mean of three independent experiments, with error bars indicating standard deviations. Significance was analyzed with Student’s *t*-test (* *p* < 0.05, ** *p* < 0.01, ns, not significant).

Viral L gene products are generated in A549 cells after infection for 12 h. Here, we detected whether UV-induced DDR prior to infection has effects on the expression of viral L genes. For this purpose, A549 cells either mock-treated or treated with UV for 4 min were infected with BoHV-1for 12 h. Then, protein levels of cell-associated viral envelope glycoprotein gD and gC were detected by Western blot using an individual monoclonal antibody. As a result, the protein expression of both gD and gC were altered by UV treatment. gD protein levels were reduced to approximately 15.21% in comparison with mock-treated controls (Figure 5A,B).

In contrast, gC protein was expressed approximately 2.68-fold higher following UV treatment (Figure 5C,D). Interestingly, UV treatment did not affect the mRNA levels of gD as detected at 12 hpi, while gC mRNA levels were significantly reduced to approximately 65.83% relative to the mock-treated controls (Figure 5E). The variation in these mRNA levels was not consistent with the alteration of individual protein levels. Since UV treatment decreases gD protein expression but not mRNA, we speculate that DDR may destabilize gD protein expression. In contrast, UV treatment reduced mRNA levels of gC but increased the protein levels, suggesting that UV-induced DDR may stabilize gC protein expression. Therefore, UV-induced DDR may differentially affect the stabilization of viral envelope glycoprotein.

### 3.4. BoHV-1 Productive Infection Has Effects on UV-Irradiation-Induced DDR Signaling γH2AX

**Figure 6:**
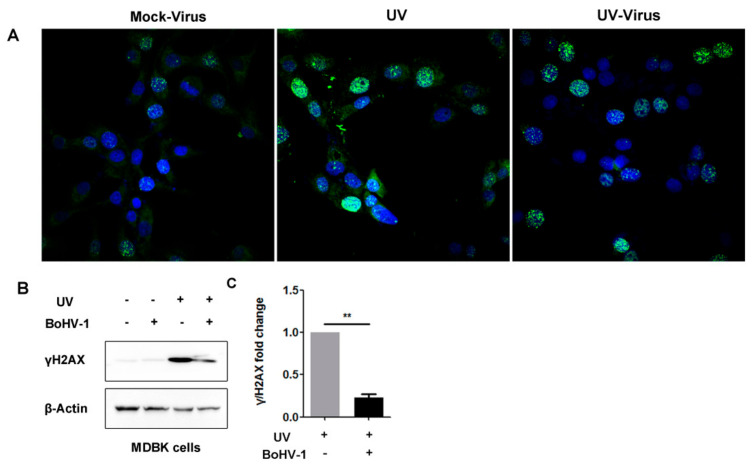
Analysis of whether BoHV-1 infection has effects on γH2AX expression and γH2AX foci formation in MDBK cells pretreated with UV light. (**A**) MDBK cells, mock-treated or treated with UV light for 4 min, were infected with BoHV-1 (MOI = 0.1) for 4 h. The cells were fixed with 4% formaldehyde and subjected to IFA assay by using γH2AX specific antibody. Images were obtained by confocal microscopy (magnification ×600). (**B**) MDBK cells, mock-treated or treated with UV light for 4 min, were infected with BoHV-1(MOI = 0.1) for 4 h. The cell lysates were prepared with RIPA lysis buffer and subjected to Western blot to detect γH2AX protein levels. (**C**) The band intensity was normalized to β-Actin, then the fold change induced by UV treatment was calculated by comparison to the mock-treated controls, which were arbitrarily set as 1. Results shown are means of three independent experiments, with error bars showing standard deviations. Significance was assessed with Student’s *t*-test (** *p* < 0.01).

It has been established that increased protein levels of γH2AX and the formation of γH2AX foci are early DDR events in response to DSBs, reviewed by [20,28]. To analyze whether BoHV-1 replication impacts UV-induced DDR, formation of γH2AX foci in both cell cultures was assessed under a confocal microscope. As can be seen in Figure 6A, clear γH2AX foci were observed in mock-treated virus-infected MDBK cells after infection for 4 h. Highlighted staining of nucleus γH2AX makes foci indiscernible in a subset of UV-treated uninfected MDBK cells. However, when these cells were subjected to UV exposure prior to virus infection, highlighted staining of nucleus γH2AX, as observed in UV-treated uninfected MDBK cells, was rarely detected, and γH2AX foci were discernible in most of the cells. Further studies by Western blotting indicated that the increased protein levels of γH2AX protein induced by UV treatment were significantly attenuated by the virus infection (Figure 6B), which were reduced to approximately 22.65% following virus infection (Figure 6C), corroborating the findings of IFA as described in Figure 6A. So, BoHV-1 infection in MDBK cells has the capacity to adjust DDR signaling as demonstrated by depletion of γH2AX protein levels stimulated by UV treatment prior to virus infection, which consequently affects the formation of γH2AX foci mainly via changing its morphology.

Similar studies were performed in A549 cells. Like what was observed in MDBK cells, clear γH2AX foci were detected in mock-treated virus-infected A549 cells after infection for 4 and 12 h, respectively. We found that the staining of nuclear γH2AX stimulated by UV treatment was significantly decreased after virus infection for 4 h but increased at 12 hpi. Clear γH2AX foci were almost indiscernible in UV-treated A549 cells following virus infection at 4 hpi, the same as is observed in UV-treated uninfected cells. 

**Figure 7:**
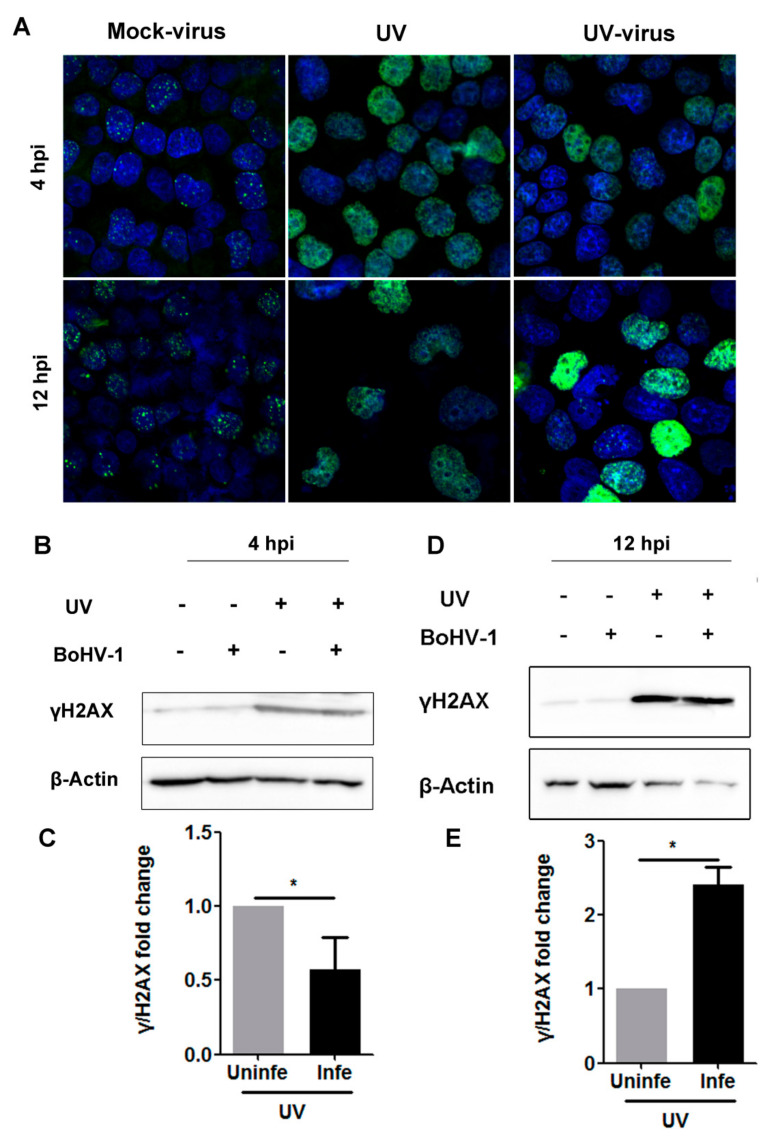
Analysis of whether BoHV-1 infection has effects on γH2AX expression and γH2AX foci formation in A549 cells pretreated with UV light. (**A**) A549 cells, mock-treated or treated with UV light for 4 min, were infected with BoHV-1 (MOI = 0.1) for 4 and 12 h, respectively. The cells were fixed with 4% formaldehyde and subjected to immunostaining with γH2AX specific antibody. Images were obtained by using confocal microscopy (magnification ×600 with 2.5 zoom). (**B**,**D**) A549 cells, mock-treated or treated with UV light for 4 min, were infected with BoHV-1(MOI = 0.1) for 4 and 12 h, respectively. The cell lysates were prepared and subjected to Western blot to detect γH2AX protein levels. Cellular protein β-Actin was detected as a protein loading control. (**C**,**E**) The band intensity was normalized to β-Actin, then the fold change induced by UV treatment was calculated by comparison to the mock-treated controls, which were arbitrarily set as 1. The results shown are means of three independent experiments, with error bars indicating standard deviations. Significance was analyzed with a Student’s *t*-test (* *p* < 0.05).

Though γH2AX foci were observed in a subset of UV-treated virus-infected cells at 12 hpi, a large amount of nuclear γH2AX with pronounced staining did not establish typical foci as observed in virus-infected controls (Figure 7A).

When the γH2AX protein levels was detected by Western blot, we found that γH2AX protein levels stimulated by UV-treatment were significantly decreased after infection for 4 h (Figure 7B), which was reduced to approximately 57.42% relative to the mock-treated uninfected controls (Figure 7C). However, after infection for 12 h, γH2AX protein levels increased by UV treatment were approximately 2.42-fold higher than the UV-treated mock-infected controls (Figure 7D,E). The variation in γH2AX protein levels attributed to virus infection corroborates the observation in the IFA assay as described in Figure 7A, suggesting that BoHV-1 replication has the capacity to adjust DDR signaling initiated by UV treatment prior to infection in human tumor cell line A549.

Taking these data together, virus infection has effects on DDR initiated by UV treatment with cell-type-dependent manners.

## 4. Discussion

The cellular DDR is a complex network of signal pathways essential for safeguarding and maintaining genome integrity. Activation of the DDR signaling network is triggered for recognition of damaged DNA and recruitment of cellular factors to repair the injuries [30]. It has been well-established that these surveillance mechanisms can also respond to the invasion of diverse DNA viruses. Vice versa, viruses have developed sophisticated strategies to limit deleterious surveillance of DDR or even to exploit components of DDR pathways for efficient replication, as reviewed in [31]. For example, HSV-1 infection exerts differential effects on varied DDR molecules, as reviewed in [32]. For example, it has been reported that HSV-1 productive infection depletes the activity and abundance of DNA-PKcs, a DDR protein, in an ICP0-dependent manner [33]. Subsequently, a report shows that HSV-1 replication is increased in cells lacking DNA-PKcs [34], suggesting that the DDR protein DNA-PKcs restricts virus infection, and ICP0-promoted degradation of DNA-PKcs is potentially beneficial to virus production. However, DNA-PKcs is recruited into AAV replication centers for the virus replication [35], giving an example that DNA-PKcs can promote AAV replication. The DDR protein ATM kinase is activated during HSV-1 productive infection and colocalizes with the virus replication compartment. Depleting ATM kinase by generating mutant cell lines or chemical inhibition of ATM reduces HSV-1 productive infection [36,37,38]. Apart from HSV-1, ATM kinase also facilitates the infection of the autonomous parvovirus minute virus of mice (MVM) [39] and human polyomavirus JC virus (JCV) [40], while MRE11/RAD50/NBS1 (MRN) complex, a multifunctional DDR machine, can bind to Adenovirus 5 (Ad5) viral genomes and activate a localized ATM, which prevents viral replication at the earliest stages [41]. Overall, a given DDR molecule may have differential effects on distinct viruses. Vice versa, different viruses may target the same DDR signaling for different purposes. Of course, distinct viruses can selectively usurp divergent DDR components to complete a life cycle, and virus infection may broadly affect the DDR network, underscoring the complexity of the interaction network. Though interplay between virus infection and numerous individual components of the DDR network has been revealed, how virus infection is affected by the overall DDR network is rarely reported in the field of virology.

Here, we are interested in whether global DDR has effects on BoHV-1 replication, as well as the mechanisms. For these studies, DDR in both MDBK cells and A549 cells is induced via UV exposure prior to virus infection. As a consequence of the cytotoxicity of UV-induced DNA damage, further cultures of 4 and 12 h after UV exposure are chosen to ensure that the cell morphology does not obviously change and cell survival is not significantly reduced (Figure 3K). Still, DDR signaling is triggered as indicated by increased levels of γH2AX protein and formation of γH2AX foci (Figure 1, Figure 2 and Figure 3). We initially determined whether global DDR induced by UV treatment affects transcription of viral IE genes using qRT-PCR to detect representative IE genes of bICP0 and bICP22.

Interestingly, the mRNA levels of both bICP0 and bICP22 were largely changed due to UV treatment with cell-type-dependent manners (Figure 4). Further investigation indicated that UV treatment prior to virus infection also affects the transcription of viral L gene gC. These data unanimously suggest that the global DDR network may influence BoHV-1 gene transcription. This mechanically corroborates the reports that both DSBs and DDR signaling can epigenetically regulate cellular gene transcription [42,43], which contributes to DSB repair. It will be an interesting question to address how these viral genes are transcriptionally affected by UV-induced DDR in the future.

Interestingly, UV-induced DDR did not affect gD mRNA levels, which cannot explain the observation that gD protein levels are significantly reduced following UV treatment (Figure 5A,E). Additionally, the reduced gC mRNA levels due to UV treatment did not support the increased protein levels (Figure 5C,E). As to these discrepancies, we suggested that UV-induced DDR may have effects on the stabilization of viral proteins via mechanisms that remain to be determined in the future. Taken together, our data suggest that UV-induced DDR can potentially regulate BoHV-1 gene transcription and viral protein stabilization.

Importantly, after infection for 4 h, the increased protein levels of γH2AX induced prior to virus infection are significantly attenuated in both MDBK and A549 cells (Figure 6 and Figure 7). However, virus infection does not always lead to depletion of γH2AX because the increased protein levels of γH2AX by UV treatment were further enhanced at 12 hpi (Figure 7A,D). It seems that BoHV-1 infection leads to either degradation or stabilization of γH2AX induced by UV treatment, with infection-stage-dependent manners. The expression of both the mRNA and protein of BoHV-1 IE genes, such as bICP0 and bICP22, is readily detected after infection for 4 h in cell culture (Figure 4 and [44]). Of note, some IE proteins of alpha herpesvirus can promote proteasome-mediated degradation of certain cellular proteins. For example, bICP0 induces protein degradation of interferon response factor 3 (IRF3) [45]. ICP0 encoded by HSV-1, a virus genetically close to BoHV-1, promotes degradation of DNA-PKcs [46]. So, it is highly possible that the viral products, such as viral proteins bICP0, generated at an early stage of infection may compromise γH2AX stabilization or promote γH2AX degradation. In view that the virus L protein can be detected at 12 hpi (Figure 5), it is possible that the viral products generated at later stages of infection can rescue the depletion of γH2AX mediated by IE products. Considering that γH2AX is a canonical DDR marker participating in DNA damage repair, a further study revealing the detailed mechanisms underlying dynamic alteration of γH2AX protein levels during virus infection is an interesting question that deserves an independent investigation in the future.

Since it has been reported that phosphorylation of H2AX extends along megabase-long domains in chromatin with both sides of the damaged DSBs, which is a marker of DNA damage [20], higher levels of γH2AX protein in UV-treated cells following virus infection (Figure 7) may indicate an exacerbation of DNA damage, which confirmed the previous report that BoHV-1 productive infection induces DNA damage [10,13]. Overwhelming accumulation of DNA damage may ultimately cause cell death, reviewed in [47,48]. Importantly, as a promising oncolytic virus, BoHV-1 has a broad antitumor spectrum, including that of human lung adenocarcinoma cells, as demonstrated in the A549 tumor xenograft mouse model [10,49,50]. Here, we speculate that the combination treatment with BoHV-1 and irradiation may result in a synergistic antitumor efficacy in vivo. Of note, the anticancer efficacy of BoHV-1 is enhanced when applied in combination with azacytidine, an FDA-approved anticancer drug [51].

However, the application of BoHV-1 in combination with irradiation, a widely used approach for cancer treatment, has not been reported. Here, our results shed light on the potential of combination with irradiation and BoHV-1 to achieve higher efficacy for cancer treatment, which deserves further studies in the future.

## 5. Conclusions

In summary, in this study, we provide evidence showing that UV-primed global DDR network differentially affected the transcription of BoHV-1 genes and stabilization of the viral protein. Vice versa, the virus productive infection has impacts on DDR as demonstrated by alterations of γH2AX protein levels and formation of γH2AX foci primed by UV treatment. To our knowledge, this is the first report regarding the interplay between UV-primed global DDR signaling and virus productive infection. Although we currently do not know how the viral gene transcription and protein expression processes are orchestrated by the global DDR signaling, these observations will extend our knowledge of the interplay between the DDR network and BoHV-1 productive infection and provide a novel perspective on understanding the virus oncolytic mechanism.

## Data Availability

The authors declare that all the data are available upon request.

## References

[1] Tikoo S.K., Campos M., Babiuk L.A. (1995). Bovine herpesvirus 1 (bhv-1): Biology, pathogenesis, and control. Adv. Virus Res..

[2] Muylkens B., Thiry J., Kirten P., Schynts F., Thiry E. (2007). Bovine herpesvirus 1 infection and infectious bovine rhinotracheitis. Vet. Res..

[3] Jones C., Chowdhury S. (2007). A review of the biology of bovine herpesvirus type 1 (bhv-1), its role as a cofactor in the bovine respiratory disease complex and development of improved vaccines. Anim. Health Res. Rev..

[4] Hodgson P.D., Aich P., Manuja A., Hokamp K., Roche F.M., Brinkman F.S., Potter A., Babiuk L.A., Griebel P.J. (2005). Effect of stress on viral-bacterial synergy in bovine respiratory disease: Novel mechanisms to regulate inflammation. Comp. Funct. Genom..

[5] Yates W.D., Babiuk L.A., Jericho K.W. (1983). Viral-bacterial pneumonia in calves: Duration of the interaction between bovine herpesvirus 1 and pasteurella haemolytica. Can. J. Comp. Med..

[6] Newcomer B.W., Cofield L.G., Walz P.H., Givens M.D. (2017). Prevention of abortion in cattle following vaccination against bovine herpesvirus 1: A meta-analysis. Prev. Vet. Med..

[7] Nandi S., Kumar M., Manohar M., Chauhan R.S. (2009). Bovine herpes virus infections in cattle. Anim. Health Res. Rev..

[8] Jones C. (2019). Bovine herpesvirus 1 counteracts immune responses and immune-surveillance to enhance pathogenesis and virus transmission. Front. Immunol..

[9] Langie S.A.S., Koppen G., Desaulniers D., Al-Mulla F., Al-Temaimi R., Amedei A., Azqueta A., Bisson W.H., Brown D., Brunborg G. (2015). Causes of genome instability: The effect of low dose chemical exposures in modern society. Carcinogenesis.

[10] Qiu W., Ding X., Li S., He Y., Zhu L. (2021). Oncolytic bovine herpesvirus 1 inhibits human lung adenocarcinoma a549 cell proliferation and tumor growth by inducing DNA damage. Int. J. Mol. Sci..

[11] Basu A.K. (2018). DNA damage, mutagenesis and cancer. Int. J. Mol. Sci..

[12] Nagaria P., Robert C., Rassool F.V. (2013). DNA double-strand break response in stem cells: Mechanisms to maintain genomic integrity. Biochim. Biophys. Acta.

[13] Zhu L., Fu X., Yuan C., Jiang X., Zhang G. (2018). Induction of oxidative DNA damage in bovine herpesvirus 1 infected bovine kidney cells (mdbk cells) and human tumor cells (a549 cells and u2os cells). Viruses.

[14] Ding X., Yuan W., Yang H., Liu C., Li S., Zhu L. (2022). β-Catenin-Specific Inhibitor, iCRT14, Promotes BoHV-1 Infection-Induced DNA Damage in Human A549 Lung Adenocarcinoma Cells by Enhancing Viral Protein Expression. Int. J. Mol. Sci..

[15] Kim R., Emi M., Tanabe K. (2005). Caspase-dependent and -independent cell death pathways after DNA damage (review). Oncol. Rep..

[16] Ferreira H.C.C., de Araujo E.N., Rosado N.C.L., Fietto J.L.R., Santos M.R., Gomes L.L., Silva L.M.N., Bressan G.C., Martins G.F., Sreevatsan S. (2021). Apoptosis in the late replication phase of bovine alphaherpesvirus 1 in experimentally infected calves. Braz. J. Microbiol..

[17] Fiorito F., Nocera F.P., Cantiello A., Iovane V., Lambiase S., Piccolo M., Ferraro M.G., Santamaria R., De Martino L. (2020). Bovine herpesvirus-1 infection in mouse neuroblastoma (neuro-2a) cells. Vet. Microbiol..

[18] Rensetti D.E., Marin M.S., Moran P.E., Odeon A.C., Verna A.E., Perez S.E. (2018). Bovine herpesvirus type 5 replication and induction of apoptosis in vitro and in the trigeminal ganglion of experimentally-infected cattle. Comp. Immunol. Microbiol. Infect. Dis..

[19] Fiorito F., Iovane V., Marullo A., Costagliola A., Granato G.E., De Martino L. (2017). 2,3,7,8-tetrachlorodibenzo-p-dioxin influences bovine herpesvirus 1 replication through upregulation of sirt3 and cytoskeletal reorganization. Vet. Res. Commun..

[20] Cardoso T.C., Rosa A.C., Ferreira H.L., Okamura L.H., Oliveira B.R., Vieira F.V., Silva-Frade C., Gameiro R., Flores E.F. (2016). Bovine herpesviruses induce different cell death forms in neuronal and glial-derived tumor cell cultures. J. Neurovirol..

[21] Hanon E., Lambot M., Hoornaert S., Lyaku J., Pastoret P.P. (1998). Bovine herpesvirus 1-induced apoptosis: Phenotypic characterization of susceptible peripheral blood mononuclear cells. Arch. Virol..

[22] Georgoulis A., Vorgias C.E., Chrousos G.P., Rogakou E.P. (2017). Genome instability and gammah2ax. Int. J. Mol. Sci..

[23] Carusillo A., Mussolino C. (2020). DNA damage: From threat to treatment. Cells.

[24] Zhu L., Yu Y., Jiang X., Yuan W., Zhu G. (2017). First report of bovine herpesvirus 1 isolation from bull semen samples in China. Acta Virol..

[25] Afroz S., Garg R., Fodje M., van Drunen Littel-van den Hurk S. (2018). The major tegument protein of bovine herpesvirus 1, vp8, interacts with DNA damage response proteins and induces apoptosis. J. Virol..

[26] Zhu L., Jones C. (2017). The high mobility group at-hook 1 protein stimulates bovine herpesvirus 1 productive infection. Virus Res..

[27] Greinert R., Volkmer B., Henning S., Breitbart E.W., Greulich K.O., Cardoso M.C., Rapp A. (2012). Uva-induced DNA double-strand breaks result from the repair of clustered oxidative DNA damages. Nucleic Acids Res..

[28] Dhuppar S., Roy S., Mazumder A. (2020). Gamma h2ax in the s phase after uv irradiation corresponds to DNA replication and does not report on the extent of DNA damage. Mol. Cell. Biol..

[29] Mah L.J., El-Osta A., Karagiannis T.C. (2010). Gammah2ax: A sensitive molecular marker of DNA damage and repair. Leukemia.

[30] Jones C. (1998). Alphaherpesvirus latency: Its role in disease and survival of the virus in nature. Adv. Virus Res..

[31] Sirbu B.M., Cortez D. (2013). DNA damage response: Three levels of DNA repair regulation. Cold Spring Harb. Perspect. Biol..

[32] Weitzman M.D., Fradet-Turcotte A. (2018). Virus DNA replication and the host DNA damage response. Annu. Rev. Virol..

[33] Smith S., Weller S.K. (2015). Hsv-i and the cellular DNA damage response. Future Virol..

[34] Lees-Miller S.P., Long M.C., Kilvert M.A., Lam V., Rice S.A., Spencer C.A. (1996). Attenuation of DNA-dependent protein kinase activity and its catalytic subunit by the herpes simplex virus type 1 transactivator icp0. J. Virol..

[35] Parkinson J., Lees-Miller S.P., Everett R.D. (1999). Herpes simplex virus type 1 immediate-early protein vmw110 induces the proteasome-dependent degradation of the catalytic subunit of DNA-dependent protein kinase. J. Virol..

[36] Schwartz R.A., Carson C.T., Schuberth C., Weitzman M.D. (2009). Adeno-associated virus replication induces a DNA damage response coordinated by DNA-dependent protein kinase. J. Virol..

[37] Lilley C.E., Carson C.T., Muotri A.R., Gage F.H., Weitzman M.D. (2005). DNA repair proteins affect the lifecycle of herpes simplex virus 1. Proc. Natl. Acad. Sci. USA.

[38] Alekseev O., Donegan W.E., Donovan K.R., Limonnik V., Azizkhan-Clifford J. (2020). Hsv-1 hijacks the host DNA damage response in corneal epithelial cells through icp4-mediated activation of atm. Investig. Ophth. Vis. Sci..

[39] Mohni K.N., Dee A.R., Smith S., Schumacher A.J., Weller S.K. (2013). Efficient herpes simplex virus 1 replication requires cellular atr pathway proteins. J. Virol..

[40] Adeyemi R.O., Landry S., Davis M.E., Weitzman M.D., Pintel D.J. (2010). Parvovirus minute virus of mice induces a DNA damage response that facilitates viral replication. PLoS Pathog..

[41] Orba Y., Suzuki T., Makino Y., Kubota K., Tanaka S., Kimura T., Sawa H. (2010). Large t antigen promotes jc virus replication in g2-arrested cells by inducing atm- and atr-mediated g2 checkpoint signaling. J. Biol. Chem..

[42] Shah G.A., O’Shea C.C. (2015). Viral and cellular genomes activate distinct DNA damage responses. Cell.

[43] Filipponi D., Emelyanov A., Muller J., Molina C., Nichols J., Bulavin D.V. (2019). DNA damage signaling-induced cancer cell reprogramming as a driver of tumor relapse. Mol. Cell.

[44] Ui A., Chiba N., Yasui A. (2020). Relationship among DNA double-strand break (dsb), dsb repair, and transcription prevents genome instability and cancer. Cancer Sci..

[45] Zhu L., Thompson J., Ma F., Eudy J., Jones C. (2017). Effects of the synthetic corticosteroid dexamethasone on bovine herpesvirus 1 productive infection. Virology.

[46] Saira K., Zhou Y., Jones C. (2007). The infected cell protein 0 encoded by bovine herpesvirus 1 (bicp0) induces degradation of interferon response factor 3 and, consequently, inhibits beta interferon promoter activity. J. Virol..

[47] Mohni K.N., Smith S., Dee A.R., Schumacher A.J., Weller S.K. (2013). Herpes simplex virus type 1 single strand DNA binding protein and helicase/primase complex disable cellular atr signaling. PLoS Pathog..

[48] Roos W.P., Kaina B. (2013). DNA damage-induced cell death: From specific DNA lesions to the DNA damage response and apoptosis. Cancer Lett..

[49] Roos W.P., Kaina B. (2006). DNA damage-induced cell death by apoptosis. Trends Mol. Med..

[50] Rodrigues R., Cuddington B., Mossman K. (2010). Bovine herpesvirus type 1 as a novel oncolytic virus. Cancer Gene Ther..

[51] Cuddington B.P., Verschoor M., Ashkar A., Mossman K.L. (2015). Enhanced efficacy with azacytidine and oncolytic bhv-1 in a tolerized cotton rat model of breast adenocarcinoma. Mol. Ther. Oncolytics.

